# Enhancing Diagnostic Accuracy of Lung Nodules in Chest Computed Tomography Using Artificial Intelligence: Retrospective Analysis

**DOI:** 10.2196/64649

**Published:** 2025-01-27

**Authors:** Weiqi Liu, You Wu, Zhuozhao Zheng, Mark Bittle, Wei Yu, Hadi Kharrazi

**Affiliations:** 1 Department of Health Policy and Management Bloomberg School of Public Health Johns Hopkins University Baltimore, MD United States; 2 Department of Research Sophmind Technology (Beijing) Co Ltd Beijing China; 3 Institute for Hospital Management School of Medicine Tsinghua University Beijing China; 4 Department of Radiology Beijing Tsinghua Changgung Hospital Tsinghua University Beijing China; 5 Department of Radiology Beijing Anzhen Hospital Capital Medical University Beijing China

**Keywords:** artificial intelligence, diagnostic accuracy, lung nodule, radiology, AI system

## Abstract

**Background:**

Uncertainty in the diagnosis of lung nodules is a challenge for both patients and physicians. Artificial intelligence (AI) systems are increasingly being integrated into medical imaging to assist diagnostic procedures. However, the accuracy of AI systems in identifying and measuring lung nodules on chest computed tomography (CT) scans remains unclear, which requires further evaluation.

**Objective:**

This study aimed to evaluate the impact of an AI-assisted diagnostic system on the diagnostic efficiency of radiologists. It specifically examined the report modification rates and missed and misdiagnosed rates of junior radiologists with and without AI assistance.

**Methods:**

We obtained effective data from 12,889 patients in 2 tertiary hospitals in Beijing before and after the implementation of the AI system, covering the period from April 2018 to March 2022. Diagnostic reports written by both junior and senior radiologists were included in each case. Using reports by senior radiologists as a reference, we compared the modification rates of reports written by junior radiologists with and without AI assistance. We further evaluated alterations in lung nodule detection capability over 3 years after the integration of the AI system. Evaluation metrics of this study include lung nodule detection rate, accuracy, false negative rate, false positive rate, and positive predictive value. The statistical analyses included descriptive statistics and chi-square, Cochran-Armitage, and Mann-Kendall tests.

**Results:**

The AI system was implemented in Beijing Anzhen Hospital (Hospital A) in January 2019 and Tsinghua Changgung Hospital (Hospital C) in June 2021. The modification rate of diagnostic reports in the detection of lung nodules increased from 4.73% to 7.23% (*χ^2^*_1_=12.15; *P*<.001) at Hospital A. In terms of lung nodule detection rates postimplementation, Hospital C increased from 46.19% to 53.45% (*χ^2^*_1_=25.48; *P*<.001) and Hospital A increased from 39.29% to 55.22% (*χ^2^*_1_=122.55; *P*<.001). At Hospital A, the false negative rate decreased from 8.4% to 5.16% (*χ^2^*_1_=9.85; *P*=.002), while the false positive rate increased from 2.36% to 9.77% (*χ^2^*_1_=53.48; *P*<.001). The detection accuracy demonstrated a decrease from 93.33% to 92.23% for Hospital A and from 95.27% to 92.77% for Hospital C. Regarding the changes in lung nodule detection capability over a 3-year period following the integration of the AI system, the detection rates for lung nodules exhibited a modest increase from 54.6% to 55.84%, while the overall accuracy demonstrated a slight improvement from 92.79% to 93.92%.

**Conclusions:**

The AI system enhanced lung nodule detection, offering the possibility of earlier disease identification and timely intervention. Nevertheless, the initial reduction in accuracy underscores the need for standardized diagnostic criteria and comprehensive training for radiologists to maximize the effectiveness of AI-enabled diagnostic systems.

## Introduction

In the rapidly evolving field of medical imaging, the integration of artificial intelligence (AI) systems is increasingly disrupting common methods of interpreting radiological images [1]. Traditionally, radiologists have been at the forefront of interpreting complex details within medical images, using their clinical expertise and experience [2]. The growing volume and complexity of medical imaging studies, however, have highlighted the limitations of human cognition [3,4]. In turn, AI systems, undeterred by the data volume, offer the potential solution for efficient, consistent, and accurate image analysis, marking a transformative change in radiological practice [5]. In practice, the China National Medical Products Administration and the US Food and Drug Administration have approved some of the AI image-assisted diagnostic systems to be marketed with the aim of improving efficiency and performance [6]. Therefore, there is an urgent need to evaluate the clinical impact before and after the implementation of AI systems in order to gather sufficient clinical evidence for their broader adoption.

In medical imaging, accurate diagnoses are crucial as such data inform clinical decisions, guide treatment plans, and significantly impact patient outcomes [7]. Radiologists are tasked with detecting abnormalities ranging from early-stage diseases to minor structural changes [8]. Any decrease in diagnostic accuracy can lead to delayed interventions, misdiagnoses, and undesired health outcomes [7]. Thus, given the impact of radiological findings on clinical decisions, improving the diagnostic accuracy of human-generated imaging reports has been a constant endeavor [9].

Several radiological studies have shown the efficacy of AI system applications in enhancing sensitivity (true positive rate) of detecting lung-related diseases [10-12], breast cancer [13,14], thyroid nodules [15], and fractures [16]. These studies, however, have been less consistent in evaluating the effect of AI systems in improving the specificity (true negative rate) of diagnosis. Lung nodule screening, a routine medical screening service, is critical for early lung cancer detection [17]. Various AI-based computer-aided diagnosis systems have been reported to substantially enhance radiologists’ performance when used as a second reader [18-20]. However, prioritizing sensitivity improvement over specificity could lead to a high number of false positives, causing unnecessary stress for patients and potentially wasting medical resources due to overuse of interventions [21].

To address the lack of studies measuring the improvement of diagnosis specificity in radiology using AI systems, this study aims to evaluate the impact of an AI-assisted lung nodule diagnostic system on the diagnostic accuracy of junior radiologists examining chest computed tomography (CT) scans. The results of this study could influence the future development of AI-assisted diagnostic systems to advance the accuracy of radiological diagnosis and treatment of lung nodules [22].

## Methods

### Study Design

This study was carried out at 2 tertiary care facilities in China, Hospital A, and Hospital C, from April 2018 to March 2022. Hospital A, in close collaboration with the Capital Medical University, offers a comprehensive radiology department consisting of 27 radiologists conducting and reviewing over 200,000 CT exams annually. Hospital C, affiliated with Tsinghua University, maintains a radiology department with over 20 radiologists. Both health care institutions provide an extensive spectrum of medical imaging services, comprehensive whole-body CT and magnetic resonance imaging scans, specialized breast imaging, as well as an array of interventional diagnostic and therapeutic procedures.

The AI-assisted diagnostic system used in this research embodies cutting-edge technology crafted to assist radiologists in the interpretation of medical images, with a specific emphasis on lung nodules. These lung nodule AI systems were developed and integrated by Care.ai [23] (Yitu, integrated into Deepwise’s framework in 2021) and Dr.Wise [24] (Deepwise) to operate at Hospital A (since January 2019) and Hospital C (since June 2021), respectively. These systems harness advanced machine learning algorithms and deep neural networks to analyze radiological images, with a primary focus on the automated detection, categorization, and characterization of abnormalities, particularly lung nodules [25].

**Figure 1:**
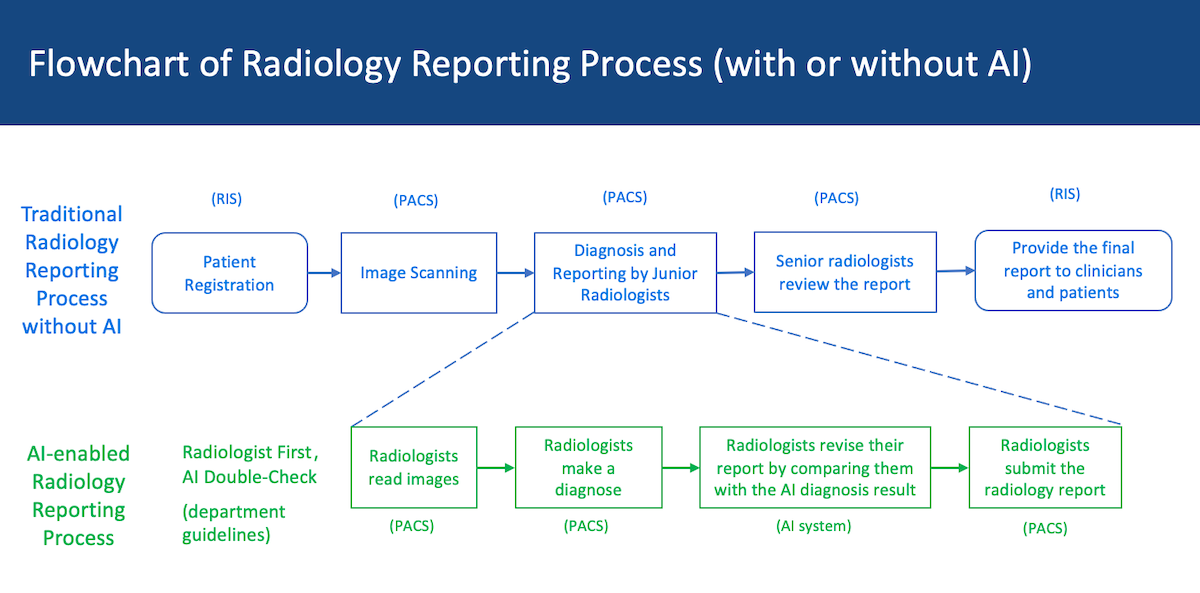
Radiologist’s diagnostic workflow with or without the artificial intelligence (AI) system. Note: AI medical devices are regulated to aid in diagnosis. All diagnoses should be made by radiologists and then checked by AI. Some radiologists may rely on the results given by the AI to double-check and modify the results. PACS: picture archiving and communication system; RIS: radiology information system.

**Figure 2:**
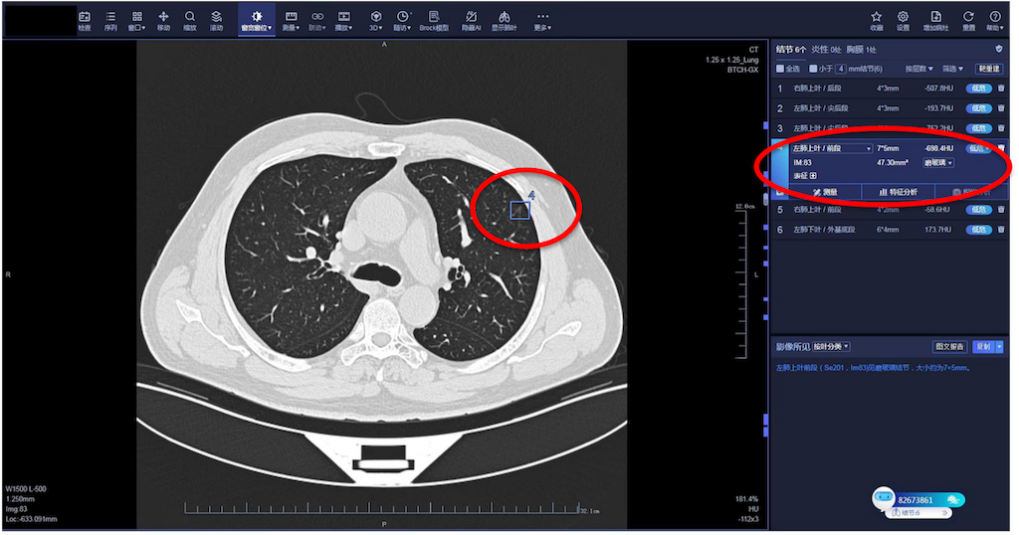
Working window of the artificial intelligence (AI) system.

The comprehensive and seamless integration of the AI tool within the clinical platform of either hospital enables a thorough assessment of lung nodules integrated into the radiologist’s diagnostic workflow (Figure 1), the AI system processes image data, generates annotations or highlights specific regions of interest, such as lung nodules, nodule location, and quantitative data, including nodule size, density, and other relevant parameters [24] (Figure 2). Junior and senior radiologists can scrutinize these findings and seamlessly integrate them into their diagnostic evaluations, thus enabling a comprehensive assessment of lung nodules (Figure 1).

To enhance efficiency and ensure accuracy, hospitals in China typically implement a 2-tiered system for radiology reporting (Figure 1). This process involves a less experienced radiologist, often a junior radiologist, who initially examines the medical images and drafts the report. This preliminary report is then passed on to a more experienced senior radiologist for further evaluation and revision. Once the report has been carefully reviewed and amended as necessary, it is finally forwarded to the physician responsible for diagnosis and also provided to the patient. In some instances, the same senior radiologist is responsible for both composing and reviewing the report. However, given that this study aimed to assess the impact of AI system introduction on the diagnostic accuracy of junior radiologists, those types of reports were excluded from the experimental sample.

We conducted a patient-specific evaluation of the reporting accuracy of junior radiologists for CT imaging. The analysis encompassed data collected from both Hospital A and Hospital C and spanned from April 2018 to March 2022. However, specific intervals, encompassing January 2019 to March 2019, January 2020 to August 2020, and April 2021 to August 2021, were excluded from the analysis due to the substantial impact of the COVID-19 pandemic [26], the implementation of the AI system, and the Spring Festival vacation on patterns of health-seeking behavior.

### Data Collection

The sample size for this study was computed with the assistance of the University of California, San Francisco calculator [27]. The aim was to detect a 30% increase (estimated risk ratio=1.3) in the efficacy of the AI intervention. To achieve 80% statistical power and significance (α=.05, 2-tailed), the study necessitated a sample size of 1594. To adhere to these criteria, the researchers adopted a systematic approach. Each month, they used Microsoft Excel for Mac (version 16.77.1), specifically using the random number sampling function to select approximately 200 patients from the complete pool of individuals who had undergone lung CT examinations at both hospitals during the study period. This methodology ensured that the selection process was unbiased and representative.

A meticulous quality control approach was followed to ensure data validity. The validation entailed the assignment of two researchers to conduct data collection and analysis, with all data undergoing a thorough anonymization process. Data were primarily collected from diagnostic reports written by junior and senior radiologists and captured in the picture archiving and communication system. The picture archiving and communication system allowed for transparent documentation of modifications made by senior radiologists to the reports initially generated by junior radiologists. Since these reports could not be directly exported from the system, photographs were taken, and the researchers summarized diagnostic report modification records. One researcher transcribed the original reports based on the photographic documentation and made adjustments pertaining to the detection of lung nodules and changes in lung lesion morphology, including modifications regarding the number, size, location, and diagnosis of lung nodules. Subsequently, another researcher thoroughly reviewed these entries to ensure accuracy. In cases of disagreement between the two entries, consultation with an expert radiologist who had more than a decade of clinical experience was sought. Due to the 2-tiered system for radiology reporting, the diagnostic results of senior radiologists were used as the reference standard to assess whether the diagnostic accuracy of junior radiologists improved after the AI system was launched. Senior radiologists set the rules for entering data on lung nodules and lung lesion morphology changes. The inclusion criteria for lung nodules included reports describing nodular shadow, punctate hyperdense shadow, and sclerotic foci. For lung lesion morphology changes, reports describing striated hyperdense shadow, patchy shadow, and ground-glass density shadow were included.

### Statistical Analysis

The primary outcome measures of this study included lung nodule detection rate, rate of missed diagnoses, rate of misdiagnoses, sensitivity, and positive predictive value. The detection rate was defined as the percentage of all patients in the study who were ultimately reported to have a lung nodule. The rate of missed diagnoses referred to the percentage of lung nodules that were not identified by the junior radiologists but were reported after review by the senior radiologists. The rate of misdiagnoses referred to instances where the junior radiologists incorrectly reported a lung nodule. Sensitivity was defined as a junior radiologist’s ability to correctly identify a true positive case, as compared with the diagnosis of a senior radiologist, which was considered the reference standard. The positive predictive value was described as the proportion of correctly identified positive cases in the diagnosis.

This study used descriptive statistics, specifically frequencies and percentages, to concisely present categorical data, particularly diagnostic outcomes. To assess statistical significance, we used the chi-square, Cochran-Armitage, and Mann-Kendall tests. The chi-square test was used to evaluate changes in both the lung nodule’s detection modification rate and detection ability as well as the lung’s morphological lesions before and after AI launch. The Cochran-Armitage and Mann-Kendall tests were used to compare the directional trend in the detection modification rate and detection ability of lung nodules and lung morphological lesions in relation to the duration of AI’s online presence. The chi-square test was used to assess relationships between categorical variables, while the Cochran-Armitage and Mann-Kendall tests addressed trend analysis. Together, these methods effectively met our study’s goals of examining intergroup relationships and temporal trends. The raw variables met the distributional assumptions of the applied tests (eg, the chi-square test does not require normality, while the Cochran-Armitage and Mann-Kendall tests are suited to nonparametric data). Therefore, raw values were used for statistical analysis. Significance was established when *P* values fell below the .05 threshold. Data analysis was carried out using R version 4.1.2 (R Development Core Team) [28], and the resulting findings were visualized using tables and graphs for enhanced interpretability.

### Ethical Considerations

This study was approved by the Institutional Review Board of the Johns Hopkins Bloomberg School of Public Health (reference number FWA 00000287). The use of data was also approved by the institutional review boards of the Tsinghua Changgung Hospital (reference number 23352-4-01) and the Beijing Anzhen Hospital (reference number 2023078X). The data used in this study were anonymous and did not contain any personally identifiable information. Furthermore, no patients can be identified in any images contained within the manuscript or its supplementary materials.

## Results

### Patient Characteristics

This study analyzed patient data collected from both hospitals before and after the implementation of the AI system. The study population included a total of 12,889 patients, with 6439 patients having an encounter with Hospital C and 6450 patients from Hospital A. The demographic characteristics of the patients are presented in Table 1.

**Table 1:** Demographic characteristics of the study population before and after the AI launch.

Variables and categories		Hospital C (n=6439)			Hospital A (n=6450)	
		Before AI^a^ (n=4830), n (%)	After AI (n=1609), n (%)	Before AI (n=1606), n (%)		After AI (n=4844), n (%)
**Age (years)**						
	<18	24 (0.5)	16 (0.99)	4 (0.25)		41 (0.85)
	18-54	1747 (36.17)	887 (55.13)	499 (31.07)		1939 (40.03)
	≥55	3059 (63.33)	706 (43.88)	1103 (68.68)		2864 (59.12)
**Gender**						
	Male	2542 (52.63)	907 (56.37)	864 (53.8)		2626 (54.21)
	Female	2288 (47.37)	702 (43.63)	742 (46.2)		2218 (45.79)
**Patient source**						
	Physical examination	514 (10.64)	231 (14.36)	5 (0.31)		188 (3.88)
	Outpatient	2187 (45.28)	451 (28.03)	1036 (64.51)		3510 (72.46)
	Emergency room	868 (17.97)	699 (43.44)	12 (0.75)		443 (9.15)
	Inpatient	1261 (26.11)	228 (14.17)	553 (34.41)		703 (14.51)
**Examination time**						
	2018.4-2018.12	1613 (33.4)	0 (0)	1606 (100)		0 (0)
	2019.4-2019.12	1608 (33.29)	0 (0)	0 (0)		1608 (33.2)
	2020.9-2021.3	1609 (33.31)	0 (0)	0 (0)		1608 (33.2)
	2021.9-2022.3	0 (0)	1609 (100)	0 (0)		1628 (33.61)

^a^AI: artificial intelligence.

### Diagnostic Accuracy

Alterations in diagnostic accuracy for the identification of lung nodules and the assessment of lung lesion morphology were measured by comparing the reports of junior and senior radiologists (Table 2). The modification rate was defined as the ratio of changes made by senior radiologists to the diagnostic reports authored by junior radiologists, divided by the total number of radiology reports. Concerning lung nodule detection, both hospitals experienced an increase in their report modification rates after the introduction of the AI system, with Hospital A showing a statistically significant rise from 4.73% to 7.23% (*χ^2^*_1_=12.15; *P*<.001) in the modification rate. Conversely, in the case of lung lesion morphology, serving as a negative control, no significant differences were observed between the two hospitals before and after the implementation of the diagnostic AI system.

**Table 2:** Diagnostic accuracy of lung nodule detection and lung lesion morphology detection before and after artificial intelligence (AI) launch.

Modified or not		Hospital C (n=6439)							Hospital A (n=6450)						
		Before AI^a^ (n=4830), n (%)	After AI (n=1609), n (%)	Chi-square (*df*)		*P* value		Before AI (n=1606), n (%)		After AI (n=4844), n (%)		Chi-square (*df*)		*P* value	
**Lung nodule**					2.27 (1)		.13						12.15 (1)		<.001
	Modified	322 (6.67)	125 (7.77)					76 (4.73)		350 (7.23)					
	Not modified	4508 (93.33)	1484 (92.23)					1530 (95.27)		4494 (92.77)					
**Lung lesion morphology**					0.01 (1)		.91						0.27 (1)		.6
	Modified	307 (6.36)	101 (6.28)					59 (3.67)		192 (3.96)					
	Not modified	4523 (93.64)	1508 (93.72)					1547 (96.33)		4652 (96.04)					

^a^AI: artificial intelligence.

### Lung Nodule Detection Ability

Alteration in lung nodule detection proficiency after the introduction of the AI system was also measured by comparing the reports of junior and senior radiologists (Table 3). At Hospital C, a notable and statistically significant rise in the detection rate was observed, increasing from 46.19% to 53.45% (*χ^2^*_1_=25.48; *P*<.001) after adopting the AI-assisted system. While a slight increase in the false negative rate and a decrease in accuracy were observed, the positive predictive value remained relatively consistent. Conversely, at Hospital A, the AI system led to a significant enhancement in the detection rate of lung nodules, elevating the rate from 39.29% to 55.22% (*χ^2^*_1_=122.55; *P*<.001). Finally, the rollout of the AI system resulted in a reduction of the false negative rate from 8.4% to 5.16% (*χ^2^*_1_=9.85; *P*=.002) but led to an increase in the false positive rate from 2.36% to 9.77% (*χ^2^*_1_=53.48; *P*<.001). Nonetheless, the detection accuracy exhibited a decline, leading to a decrease in the positive predictive value, the rate from 96.17% to 92.29% (*χ^2^*_1_=11.41; *P*<.001).

**Table 3:** Lung nodule detection ability before and after artificial intelligence (AI) launch.

Hospital	Diagnostic method	Detection rate, %	False negative rate, %	False positive rate, %	Accuracy, %	Positive predictive value^a^, %
Hospital C	CT^b^ examination	46.19	10.98	2.96	93.33	96.27
	AI-assisted^c^ system + CT examination	53.45	11.28	3.74	92.23	96.46
Hospital A	CT examination	39.29	8.4	2.36	95.27	96.17
	AI-assisted system + CT examination	55.22	5.16	9.77	92.77	92.29

^a^Positive predictive value: The proportion of correctly identified positive cases in the diagnosis.

^b^CT: computed tomography.

^c^AI-assisted: artificial intelligence–assisted.

### Subgroup Analysis of Different AI Launch Times

To examine the patterns of diagnostic accuracy and detection ability of the AI system over time, we conducted subgroup analyses of different AI launch times (Table 4). Regarding lung nodules, the percentage of modified diagnoses increased from 7.21% in the first year to 8.40% in the second year but then decreased to 6.08% in the third year. Based on the Mann-Kendall test, the *z* score of –1.25 indicated a nonsignificant trend (*P*=.21). As for lung lesion morphology, the percentage of modified diagnoses remained relatively stable, with a slight increase to 4.42% in the third year. The *z* score of 0.83 also indicated a nonsignificant trend (*P*=.41). Most diagnoses remained unchanged during the 3 years following the launch of the AI system.

**Table 4:** Time trend analysis of lung nodule diagnostic accuracy from different artificial intelligence (AI) launch times at Hospital A.

Modified or not		1 year after AI^a^ launch (n=1608), n (%)	2 years after AI launch (n=1608), n (%)	3 years after AI launch (n=1628), n (%)	*z*^b^ score		*P* value	
**Lung nodule**						–1.25		.21
	Modified	116 (7.21)	135 (8.4)	99 (6.08)				
	Not modified	1492 (92.79)	1473 (91.6)	1529 (93.92)				
**Lung lesion morphology**						0.83		.41
	Modified	62 (3.86)	58 (3.61)	72 (4.42)				
	Not modified	1546 (96.14)	1550 (96.39)	1556 (95.58)				

^a^AI: artificial intelligence.

^b^The test statistic for the Mann-Kendall test.

Alterations in lung nodule detection capability were measured over 3 years after the integration of the AI system (Table 5). The detection rates for lung nodules displayed a slight increase from 54.6% to 55.84%. In parallel, the false negative rates decreased from 7.06% to 4.07%. Nevertheless, the false positive rates exhibited fluctuations, reaching a peak of 13.33% in the second year. Despite these variations, the overall accuracy remained notably high, with a slight increase from 92.79% to 93.92%. Similarly, the positive predictive value demonstrated a comparable trend, with a minor decline in the second year.

**Table 5:** Time trend analysis of lung nodule detection ability from different artificial intelligence (AI) launch times at Hospital A.

Examination time	Detection rate, %	False negative rate, %	False positive rate, %	Accuracy, %	Positive predictive value^a^, %
1 year after AI^b^ launch	54.6	7.06	7.4	92.79	93.79
2 years after AI launch	55.22	4.39	13.33	91.6	89.84
3 years after AI launch	55.84	4.07	8.62	93.92	93.36

^a^Positive predictive value: The proportion of correctly identified positive cases in the diagnosis.

^b^AI: artificial intelligence.

## Discussion

### Enhanced Detection of Lung Nodules

Our study noted a substantial improvement in the detection rate of lung nodules following the implementation of the AI system. This improvement can be attributed to the AI’s capability to augment radiologists’ ability to spot small nodules that might indicate an underlying disease. AI algorithms are designed to methodically examine a wide range of medical images, thereby highlighting potential abnormalities that the human eye might miss. Furthermore, small lung nodules are often linked to early-stage diseases, which provide additional time for effective interventions, thus increasing the value of AI diagnostic systems in improving the detection of lung nodules in CT scans as well as the overall outcome of such patients [10].

### Initial Decrease in Diagnostic Accuracy

Interestingly, compared with the diagnostic results of radiologists without AI assistance, our study observed a decline in the diagnostic accuracy of lung nodules within 3 years of the introduction of the AI system, mainly due to a significant rise in misdiagnosis rates. This intriguing observation calls for a more detailed investigation into the complex relationship between AI technology and radiologists’ clinical decision-making. The increase in misdiagnosis rates could be due to the following reasons. First, the inherent characteristics of lung nodules present challenges for accurate detection. Specifically, CT imaging can be difficult when nodules are small, faint, or located in endobronchial or hilar regions [29]. In addition, pulmonary vasculature and artifacts were two of the main causes of false positive findings [30]. In addition, varying clinical attitudes of radiologists on reporting very small, and often microscopic, nodules may affect the detection accuracy. Radiologists may differ in their evaluation of clinical significance, resulting in varied reporting practices [31-33]. Furthermore, while the AI system is proficient in detecting nodules, it may also lead to a surge in cases with minimal clinical relevance, contributing to higher misdiagnosis rates. In order to reduce the false positives brought about by the introduced AI system, it is necessary to take comprehensive measures to maximize the effectiveness of the AI system in the future. In terms of algorithms, some algorithms have attempted to use a 2-step strategy, with the first step being the detection of candidate nodules with high specificity, followed by a false-positive reduction step in recent years [34]. For radiologists, before formally applying AI in the clinic, radiologists should receive training [35,36], which requires standardized diagnostic criteria to avoid diagnostic alterations due to differing attitudes of radiologists towards microscopic nodules. Meanwhile, promoting the standardized application of Chinese expert consensus on the diagnosis and treatment of pulmonary nodules (2024) in clinical practice is essential [6].

### Long-Term Use and Temporal Trends

Our study revealed intriguing temporal trends in the diagnostic accuracy of lung nodules. Over time, we noticed a steady improvement in accuracy, indicating a dynamic process of adaptation and refinement. Several factors could potentially explain this phenomenon. One likely reason could be the ongoing optimization of AI algorithms through machine learning and feedback from actual clinical use [37,38]. As AI systems gain more experience in interpreting medical images and learning from radiologists’ diagnostic patterns, their performance becomes increasingly refined. Furthermore, the growing familiarity and acceptance of AI systems among physicians could enhance their ability to work in tandem with these tools, thereby leading to improved diagnostic accuracy [39,40]. These pieces of evidence suggest that the AI system is expected to identify the unique features of lung nodules that differentiate them from other anatomies, as well as develop the capability for 3D reconstruction.

### Strengths and Limitations

This study has several strengths. First, we conducted a statistical analysis of 12,889 diagnostic reports before and after the implementation of the AI system in 2 tertiary hospitals in China. The large sample size enabled us to draw reliable conclusions about the impact of the AI system on radiologists’ performance. Second, we specifically selected the widely used lung nodule AI system to analyze detection rates and diagnostic accuracy, aiming to improve the quality of care and patient diagnosis in a real-world scenario. Finally, to evaluate the long-term application of the AI system, we included a random sample of diagnostic reports spanning 4 years, thus allowing us to conclude the temporal trends in the ongoing optimization process.

This study also has limitations. First, the research was conducted exclusively in 2 tertiary hospitals situated in Beijing, China. It is essential to recognize that junior radiologists, despite having relatively limited diagnostic experience, boast a high educational background and medical proficiency. Therefore, the influence detected by the introduction of the AI system in our study might not be generalizable to other institutions, specifically primary care settings. Furthermore, it is pertinent to acknowledge that our comparative benchmark was based on the diagnostic outcomes of senior radiologists. While the diagnoses rendered by senior radiologists are undoubtedly a robust reference, it is important to acknowledge that distinctions between clinical and pathological assessments may exist, and senior radiologists’ evaluation and revision can be influenced by the implementation of AI systems too.

For future investigations, it is advisable to contemplate the collection of prospective data and the incorporation of pathological results to enable a more comprehensive evaluation.

### Conclusion

In summary, the integration of AI systems has yielded significant enhancements in lung nodule detection rates, particularly in the case of small nodules. This advancement, however, has been accompanied by a temporary decrease in diagnostic accuracy, primarily attributed to increased misdiagnosis rates, potentially arising from the influence of varying diagnostic criteria following the integration of AI systems and the performance of AI proficiency in detecting tiny lung nodules while ignoring clinical significance. Nonetheless, our research reveals encouraging trends over time, with diagnostic precision gradually ameliorating. This improvement can be ascribed to the continual refinement of AI algorithms and more effective collaboration among radiologists. Overall, our study underscores the promising role of AI in clinical settings, thus presenting opportunities for early disease identification and personalized patient care.

## Abbreviations

AI: artificial intelligence
CT: computed tomography

## Data Availability

The datasets generated during and/or analyzed during this study are not publicly available due to restricted usage imposed by the patient privacy protection policy from Beijing Anzhen Hospital (Hospital A) and Tsinghua Changgung Hospital (Hospital C). The dataset applied in this study can be accessed with permission from the institutional review boards of Hospital A and Hospital C.
